# Anti-Fatigue and Antioxidant Activity of the Polysaccharides Isolated from *Millettiae speciosae* Champ. Leguminosae

**DOI:** 10.3390/nu7105422

**Published:** 2015-10-21

**Authors:** Xiao-Ning Zhao, Jia-Li Liang, Han-Bin Chen, Ye-Er Liang, Hui-Zhen Guo, Ze-Ren Su, Yu-Cui Li, Hui-Fang Zeng, Xiao-Jun Zhang

**Affiliations:** 1 The First Affiliated Hospital of Chinese Medicine, Guangzhou University of Chinese Medicine, Guangzhou 510405, China; 15017592619@163.com (X.-N.Z.); chenhanbin99@126.com (H.-B.C.); 2 Department of Chinese Materia Medica, Guangdong Xinxing Junior College of the Traditional Chinese Medicine, Xinxing 527400, China; 3 School of Chinese Materia Medica, Guangzhou University of Chinese Medicine, Guangzhou 510006, China; jackieleung08@sina.cn (J.-L.L.); mb55801@umac.mo (Y.-E.L.); guohuizhen88@126.com (H.-Z.G.); suziren@gzhtcm.edu.cn (Z.-R.S.); liyucui@gzucm.edu.cn (Y.-C.L.)

**Keywords:** *Millettiae speciosae* Champ., polysaccharides, anti-fatigue activity, antioxidant activity, physical performance, biochemical indicators

## Abstract

*Millettiae speciosae* Champ. Leguminosae (MSC), is a well-known Chinese herb traditionally used as food material and medicine for enhancing physical strength. Our preliminary study found that the aqueous extract of this herb (MSE) had an anti-fatigue effect. In this paper, we further separated MSE into total polysaccharides (MSP) and supernatant (MSS) by alcohol precipitation, and explored which fraction was active for its anti-fatigue effect. Mice were orally administered with MSP or MSS at the doses of 200, 400, and 800 mg/kg for 20 days and the anti-fatigue effect was assessed by exhaustive swimming exercise (ESE). The biochemical parameters related to fatigue after ESE and the *in vitro* antioxidant activity of active fraction were determined. Our results showed that MSP, instead of MSS, significantly extended the swimming time to exhaustion (*p* < 0.05), indicating that MSP is responsible for the anti-fatigue effect of MSE. In addition, MSP treatment increased the levels of glucose (Glu) and muscle glycogen, whereas it decreased the accumulations of blood urea nitrogen (BUN) and lactic acid (Lac). Moreover, ESE increased the levels of creatine phosphokinase (CK), lactic dehydrogenase (LDH), and malondialdehyde (MDA) but reduced superoxide dismutase (SOD) and glutathione (GSH) in plasma. In contrast, MSP inhibited all the above changes relating to fatigue. Furthermore, an *in vitro* antioxidant test revealed that MSP dose-dependently scavenged ·OH and DPPH free radicals. Taken together, these findings strongly suggested that MSP was able to alleviate physical fatigue by increasing energy resources and decreasing accumulation of detrimental metabolites. The antioxidant activity may crucially contribute to the observed anti-fatigue effect of MSP.

## 1. Introduction

Physical fatigue refers to the condition that due to over-exercise, the body fails to maintain its specific physiological level or predetermined exercise intensity [1,2]. Among the theories explaining the mechanisms of physical fatigue, energy exhaustion, and radical accumulation have attracted most interest. Recent decades have witnessed health scholars and athletic physiologists who have paid more and more attention to seeking natural anti-fatigue remedies. A number of traditional Chinese herbs, such as Acanthopanax senticosus, are found to have great potential, since they not only improve athletic ability, postpone fatigue, accelerate the elimination of fatigue in human beings, but also have few side effects [3,4,5].

*Millettiae speciosae* Champ. (MS) belongs to the Leguminosae family which is indigenous to Guangdong Province, China. As a traditional Chinese medicine, MS functions to reinforce physical strength and tonify deficiency [6]. We have previously shown for the first time that MS Extract (MSE) had significant anti-fatigue activity [7], but which fraction of MS is active remains unclear. Previous studies have shown that polysaccharides, commonly extracted from plants such as Cordyceps [8] and Ginseng [9], are the main bioactive fractions against fatigue. Thus, the present study was designed to extend our previous work by separating MSE into total polysaccharides (MSP) and supernatant (MSS), with an aim to identify the anti-fatigue constituents.

It is well known that depletion of energy, accumulation of detrimental metabolites, and production of free radicals all contribute to fatigue. At the start of exercise, energy sources such as Glu and glycogen are used first and may soon become exhausted [10], causing the production and accumulation of metabolic products including lactic acid and ammonia in the body [11]. Strenuous exercise causes oxidative stress, leading to an imbalance between reactive oxygen species (ROS) production and antioxidant defense. Excessive ROS cause oxidative damage to the cytomembrane of myocyte, leading to leakage of cytoplasm [3]. Thus, oxidative stress plays an important role in the pathophysiology of fatigue caused by exhaustive exercises [10]. On the other hand, antioxidants can alleviate fatigue by counteracting inter- or intra-cellular oxidative substrates [4].

Therefore, to elucidate the underlying anti-fatigue mechanisms, biochemical parameters related to fatigue were measured. Specifically, the levels of glucose (Glu), gastrocnemius muscle glycogen, blood urea nitrogen (BUN), and lactic acid (Lac) were determined to monitor energy consumption and accumulation of detrimental metabolites. Lactic dehydrogenase (LDH) and creatine phosphokinase (CK) were measured to assess cell damage. Malondialdehyde (MDA), superoxide dismutase (SOD), and glutathione (GSH) were assayed to reveal the intracorporal oxidative damage and anti-oxidative capacity. Also, the *in vitro* scavenging activity to hydroxyl and DPPH radicals were examined to confirm the antioxidant properties of active fraction.

## 2. Experimental Section

### 2.1. Plant Material and Preparation

The rhizome of *Millettiae speciosae* Champ. Leguminosae was purchased from the Chinese herbal drug market in Guangdong Province, and identified by Su Zi Ren in January 2013. A voucher specimen (No. 201301290109) was deposited in the First Affiliated Hospital of Chinese Medicine, Guangzhou University of Chinese Medicine.

Total polysaccharides (MSP) and supernatant (MSS) were prepared as follows: the crude herbs were extracted with boiling water (1:15, wt/wt) for 3 h. Subsequently, 95% ethanol was added to the aqueous extract up to 80% to precipitate the polysaccharides. After centrifugation, supernatant was vacuum-dried at 60 °C, to yield the MSS. While the precipitant was re-dissolved by distilled water, de-proteined by adding 15% trichloroacetic acid and then lyophilized to obtain MSP. Both MSP and MSS were stored at 4 °C.

### 2.2. Determination of Total Polysaccharides in MSP and MSS

Concentrations of total polysaccharides from MSP and MSS were determined by sulfuric acid-anthrone method using glucose as a standard following the previous method [12].

### 2.3. Chemicals and Reagents

Commercial kits to determine CK, LDH, SOD, MDA, and GSH were obtained from Nanjing Jiancheng Bioengineering Institute (Nanjing, Jiangsu, China). 2,2-diphenyl-1-picrylhydrazyl (DPPH) was purchased from Shanghai Macklin Biochemical Co. Ltd (C10014362) (Shanghai, China) and crystal violet was obtained from Tianjin City, Damao Chemical Reagent Factory (20140120) (Tianjin, China). All other chemicals were of analytical grade unless stated specifically.

### 2.4. Animals and Treatments

Male Kunming mice, weighted 15–17 g, were obtained from the Laboratory Animal Centre, Guangzhou University of Chinese Medicine (No. 44007200002880). All experiments were carried out according to guidelines of the National Institutes of Health Guide for Care and Use of Laboratory Animals [13], and were approved by the Animal Care & Welfare Committee of Guangzhou University of Chinese Medicine (No. dspf 2014009). Mice were maintained under a controlled environment (12 h light/dark cycle, temperature 22 ± 2 °C). Food and water were available *ad libitum*. Body weights were recorded daily during the 20 day experimental period.

### 2.5. Exhaustive Swimming Exercise (ESE) Test

The exercise performance was evaluated by an ESE test as described previously with minor modification [14]. Briefly, 72 mice were randomly divided into the following eight groups and exposed to the ESE. Group 1: control-mice treated with saline; Group 2: positive-mice treated with 800 mg/kg spirulina; Group 3–5: mice treated with 200, 400, or 800 mg/kg MSP for low-, middle-, and high-dose respectively; Group 6–8: mice treated with 200, 400, or 800 mg/kg MSS for low-, middle-, and high-dose respectively. Saline or the corresponding agents were administered orally (9:00 am) to mice for 20 consecutive days. The mice were made to swim for 5 min once per week without loads as adaptive training before test.

One hour after the final gavage treatment, the ESE test involved mice carrying constant loads of 5% of their body weight to test the endurance time. Records were made from the beginning of the test until exhaustion, determined by loss of coordinated movements and failure to return to the surface within 10 s.

### 2.6. Mechanistic Study of the Anti-Fatigue Activity

Ninety-six mice were randomly divided into six groups and experimental drugs were administered orally for 20 consecutive days. Group 1: untreated control-mice unexposed to ESE but received saline; Group 2: vehicle-mice exposed to ESE and treated with saline; Group 3: positive-mice exposed to ESE and treated with 800 mg/kg spirulina; Group 4–6: MSP-mice exposed to ESE and treated with 200, 400, or 800 mg/kg MSP for low-, middle-, and high-dose respectively.

After the final treatment, mice were forced to swim for 30 min without loads [15]. Blood was withdrawn from orbital venous plexus and centrifuged at 3000 rpm at 4°C for 10 min. Gastrocnemius were taken from the right legs of the mice. Levels of glucose (Glu), blood urea nitrogen (BUN), creatine phosphokinase (CK), lactic dehydrogenase (LDH), and Lac were measured by using Cosba 8000 automatic biochemistry analyzer (Roche, Germany). Levels of superoxide dismutase (SOD), glutathione (GSH), malondialdehyde (MDA), and muscle glycogen were measured by using commercially available kits from Nanjing Jiancheng Bioengineering Institute.

### 2.7. In Vitro Antioxidant Experiment

#### 2.7.1. ·OH Scavenging Activity

The ·OH scavenging activity was carried out using a modified method of crystal violet staining [16]. In brief, the reaction mixture contained 1.4 mL of 2 × 10^−5^ M crystal violet solution, 1.0 mL of 5 mM FeSO_4_, 0.4 mL of 1% H_2_O_2_, 1.0 mL of 0.1 M citric acid-sodium citrate solution and 0.5 mL of varied concentrations of MSP or MSS (50, 100, 200 μg/mL). Vitamin C (Vc, 200 μg/mL) was used as standard reference. The mixture was shaken vigorously and left to stand for 10 min. The absorbance of the hydroxylated crystal violet complex was measured at 588 nm using a Shimadzu UV-2550 spectrophotometer. All experiments were carried out in triplicate. The ·OH scavenging rate (HSR) was calculated with the equation:

$$\text{HSR}\left(\text{\%}\right)=\frac{As-Ab}{A\text{o}-Ab}\times 100\%$$

where *As* is the absorbance of the samples, *Ao* the absorbance of the negative control (without Fe^2+^ and H_2_O_2_), and *Ab* the absorbance of Vc.

#### 2.7.2. DPPH Scavenging Activity

The DPPH scavenging activities of MSP and MSS were analyzed as described previously with minor modification [17]. Briefly, DPPH has an absorption band at 517 nm, which disappears upon reduction by antioxidant. An aliquot of the ethanol (2 mL) containing various doses of MSP or MSS (50, 100, 200 μg/mL) was added to 2 mL of freshly prepared DPPH solution (25 mg/mL in ethanol). Vc at 200 μg/mL was used as standard reference. The mixture was shaken vigorously and left to stand for 20 min. The optical density of the solution was measured at 517 nm using a Shimadzu UV-2550 spectrophotometer. All experiments were carried out in triplicate. The DPPH scavenging rate (DSR) was calculated with the equation:

$$\text{DSR}\left(\text{\%}\right)=\frac{1-\left(As-Aj\right)}{Ac}\times 100\%$$

where *As* is the absorbance of the samples, *Aj* the absorbance of the negative control (without DPPH), and *Ac* the absorbance of Vc.

### 2.8. Statistical Analysis

All data were expressed as mean ± SD or SEM for *n* = 8 mice per group. Statistical differences among groups were analyzed using one-way analysis of variance (ANOVA), and the linear by linear association for dose-effect trend analysis using SPSS software (SPSS Inc., Chicago, IL, USA) followed by Tukey’s HSD test as a post hoc comparison. A value of *p* < 0.05 was considered statistically significant.

## 3. Results

### 3.1. Quantification of Total Polysaccharides

Our preliminary study revealed that MSE effectively alleviated physical fatigue [7]. In this research, we further separated MSE into MSP and MSS using the method of ethanol precipitation, a classic method used to purify or concentrate polysaccharides from aqueous solutions by adding ethanol as an antisolvent [7,9]. The content of total polysaccharides in MSP, determined by classic sulfuric acid-anthrone colorimetric test, was 62.13% ± 0.65%. Since there still existed some monosaccharides and oligosaccharides in MSS which could not be completely precipitated even by high concentrations of ethanol, the total saccharides content of MSS was 11.09% ± 0.52%. Thus, the polysaccharides content of MSP was at least five times that of MSS.

### 3.2. Effect of MSP and MSS on Body Weights of Mice

The body weights of mice were measured for 20 consecutive days during the experiment. As shown in Table 1, neither the initial nor final body weights in MSP or MSS groups were of significant difference compared with control group (*p* > 0.05). Thus, MSP and MSS had no significant influence on the body weights of the mice.

**Table 1:** The body weights of mice in all treatment groups (x ± SD).

Group	*N*	Dose(mg/kg/day)	Initial(g)	Final(g)
Control	8	0	16.3 ± 1.1	28.7 ± 1.4
positive	8	800	16.4 ± 1.8	28.2 ± 0.9
MSP	8	200	16.2 ± 0.9	29.0 ± 1.3
	8	400	16.2 ± 1.0	30.0 ± 1.8
	8	800	16.4 ± 1.2	29.5 ± 2.2
MSS	8	200	16.3 ± 1.4	29.9 ± 1.4
	8	400	15.8 ± 0.9	28.1 ± 1.7
	8	800	16.1 ± 1.1	30.0 ± 1.6

### 3.3. Effects of MSP and MSS on the Performance of Mice in ESE Test

**Figure 1:**
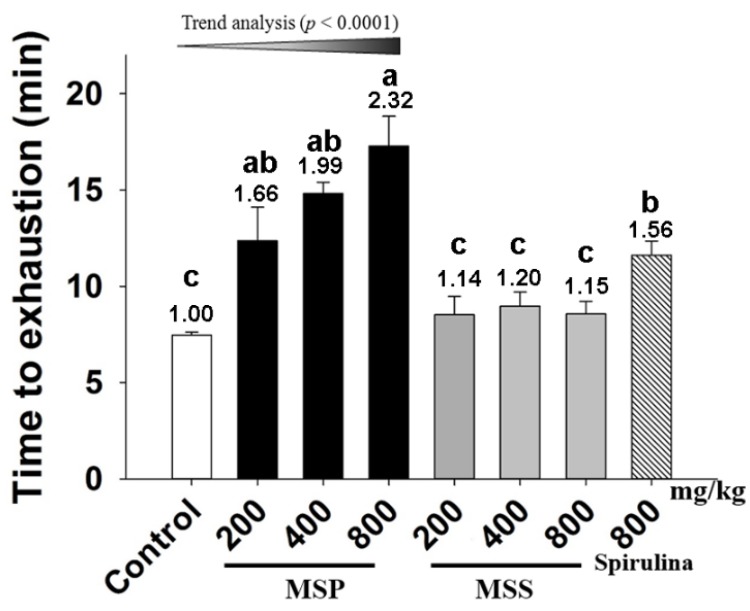
Effect of *Millettiae speciosae* Champ fractions on exhaustive swimming exercise (ESE) test. Control group (saline, open column), *Millettiae speciosae* Champ. total polysaccharides (MSP)-treated group (200, 400, 800 mg/kg, black column), MSS-treated group (200, 400, 800 mg/kg, grey column), positive group (spirulina, 800 mg/kg, twill column). Data are mean ± standard error of the mean (SEM) for *n* = 8 mice per group. Bars with different letters (a,b,c) denote significantly different at *p* < 0.05. Numbers above the bars are the fold of increase *vs.* Control.

Exercise endurance is an important variable in assessing anti-fatigue effects. We evaluated the effect of MSP and MSS on the exercise endurance of the mice by an ESE test. As shown in Figure 1, the endurance time in the control, spirulina, MSP-200, MSP-400, and MSP-800 mg/kg groups were 11.62, 12.37, 14.82, and 17.28 min, respectively. Compared with that of the control group, the endurance time periods of spirulina, MSP-400, and MSP-800 mg/kg groups are 1.56 (*p* = 0.017), 1.99 (*p* = 0.000), and 2.32 fold (*p* = 0.009) longer, respectively. Trend analysis revealed a dose-dependent increase in the three MSP group. In contrast, all the three doses of MSS groups showed no significant differences (*p* > 0.05) compared with the control group.

### 3.4. Effects of MSP on Biochemical Parameters Related to Fatigue in Mice

The above results demonstrated that MSP instead of MSS possessed anti-fatigue activity. Therefore, we further analyzed a possible mechanism by determining the related biochemical markers in mice after 30 min of forced swim.

#### 3.4.1. Effects of MSP on Energy Consumption

**Figure 2:**
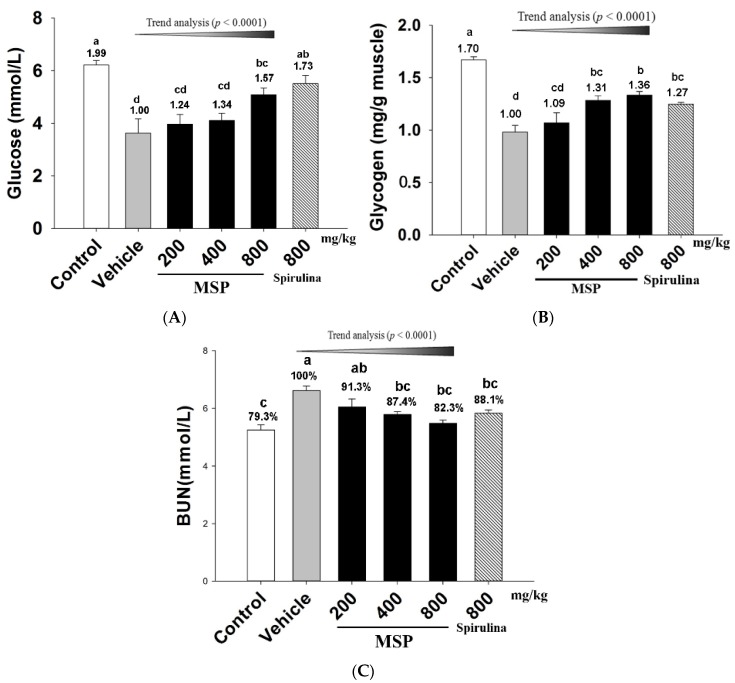
Effect of *Millettiae speciosae* Champ. total polysaccharides (MSP) on the level of plasma Glu (**A**); muscle glycogen (**B**) and blood urea nitrogen (BUN) (**C**) in mice after swimming for 30 min. Control group (open column), vehicle group (grey column), MSP-treated group (200, 400, 800 mg/kg/day, black column), spirulina group (800 mg/kg/day, twill column). Data are mean ± standard error of the mean (SEM) for *n* = 8 mice per group. Bars with different letters (a, b, c, d) denote significantly different at *p* < 0.05. Numbers and percentages above the bars are the fold or percentage to the vehicle treatment.

As shown in Figure 2, exposure to the forced swim led to a decrease in plasma Glu (Figure 2-A) and muscle glycogen (Figure 2-B) but an increase in BUN (Figure 2-C). A significant difference was found between the vehicle and control groups. However, spirulina and MSP (800 mg/kg) significantly increased Glu by 72.7% and 57.3% respectively when compared with the vehicle treatment. The muscle glycogen content (Figure 2-B) of MSP groups at the doses of 400 and 800 mg/kg were significantly elevated when compared to that of the vehicle group (*p* < 0.05). Although the glycogen of the MSP 200 mg/kg group was also increased by 9.02% compared with the control group, the difference was not significant (*p* > 0.05). Trend analysis revealed a dose-dependent increase in Glu and muscle glycogen in MSP groups (*p* < 0.0001). The serum level of BUN is an important indicator of fatigue after intense exercise. As shown in Figure 2-C, the MSP-treated groups (at the doses of 400 and 800 mg/kg) showed significantly lower BUN compared with the vehicle treatment (*p* < 0.05). Trend analysis also revealed a dose-dependent decrease in the three MSP groups.

#### 3.4.2. Effect of MSP on the Accumulation of Metabolites

**Figure 3:**
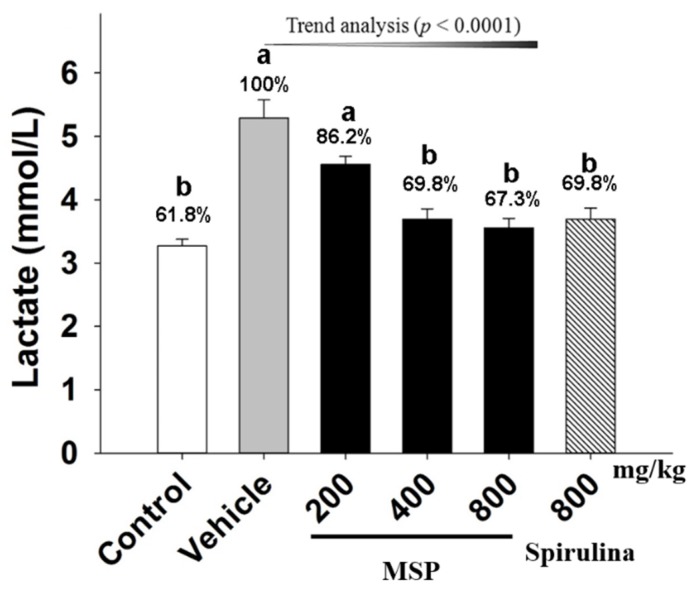
Effect of *Millettiae speciosae* Champ. total polysaccharides (MSP) on the levels of plasma Lac in mice after swimming for 30 min. Control group (open column), vehicle group (grey column), MSP-treated groups (200, 400, 800 mg/kg/day, black column), spirulina group (800 mg/kg/day, twill column). Data are mean ± standard error of the mean (SEM) with *n* = 8 mice per group. Bars with different letters (a, b) are significantly different at *p* < 0.05. Percents shown above the bars indicate the percentage to the vehicle treatment.

Lac, an important metabolite, is known to accumulate during intensive prolonged exercise. As shown in Figure 3, the results showed that the level of Lac was elevated in mice after forced swimming, with the vehicle group having higher level of Lac than that of the control. Spirulina significantly attenuated the content of Lac by 30.15% when compared to the vehicle treatment. Meanwhile, MSP at the doses of 200, 400, and 800 mg/kg decreased the content of Lac by 13.84%, 30.15%, and 32.72%, respectively.

#### 3.4.3. The Protective Effects of MSP

**Figure 4:**
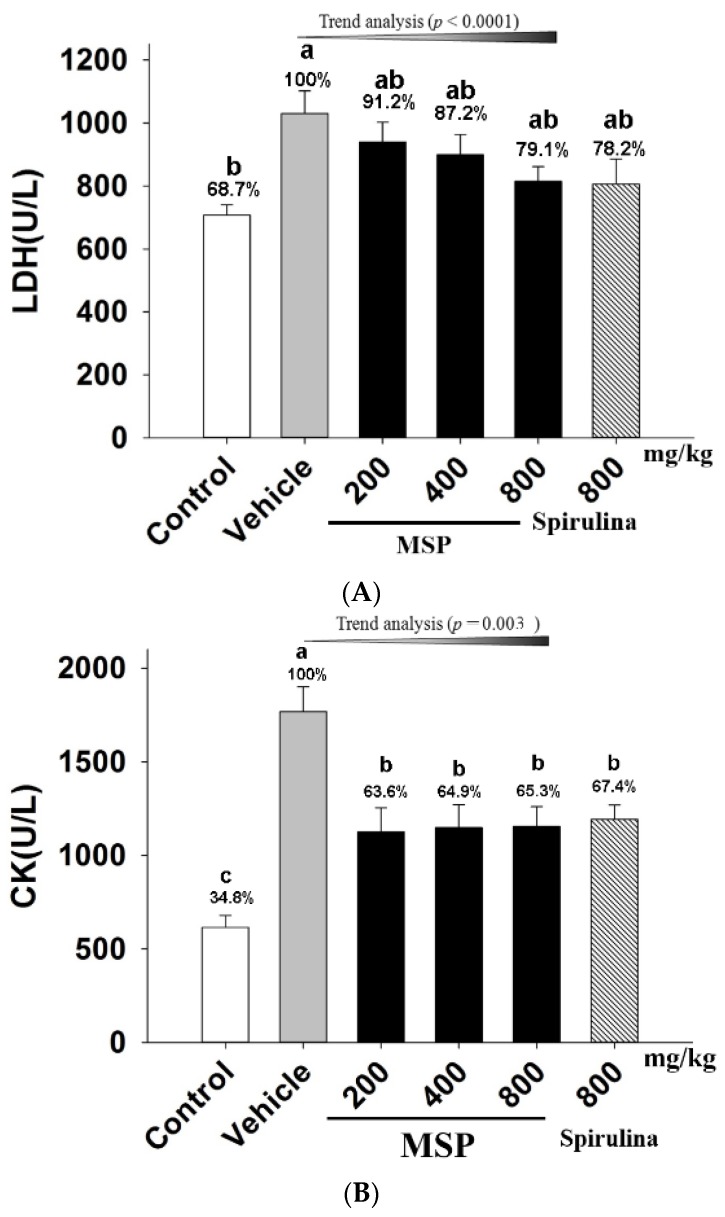
Effects of MSP on plasma lactic dehydrogenase (LDH) (**A**) and creatine phosphokinase (CK) (**B**) in mice after swimming for 30 min. Control group (open column), vehicle group (grey column), MSP-treated groups (200, 400, 800 mg/kg/day, black column), spirulina group (800 mg/kg/day, twill column). Data are mean ± standard error of the mean (SEM) with *n* = 8 mice per group. Bars with different letters (a, b) denote significant difference at *p* < 0.05. Percents shown above the bars represent percentage of the vehicle treatment.

As shown in Figure 4-A and 4-B, when mice exposed to forced swim, the plasma LDH and CK of the vehicle treatment group were significantly increased when compared with the control animals. However, serum LDH level of the MSP groups were significantly reduced by 8.8%, 12.8%, and 20.9%, compared to the vehicle group. In addition, MSP at the doses of 200, 400, and 800 mg/kg significantly decreased the plasma CK by 36.40%, 35.12%, and 34.71% respectively when compared with the vehicle treatment (Figure 4-B). Trend analysis also revealed significant correlation between increased doses of MSP and decreased plasma LDH (*p* < 0.0001), as well as decreased plasma CK (*p* < 0.003).

#### 3.4.4. *In Vivo* Antioxidative Activity of MSP

**Figure 5:**
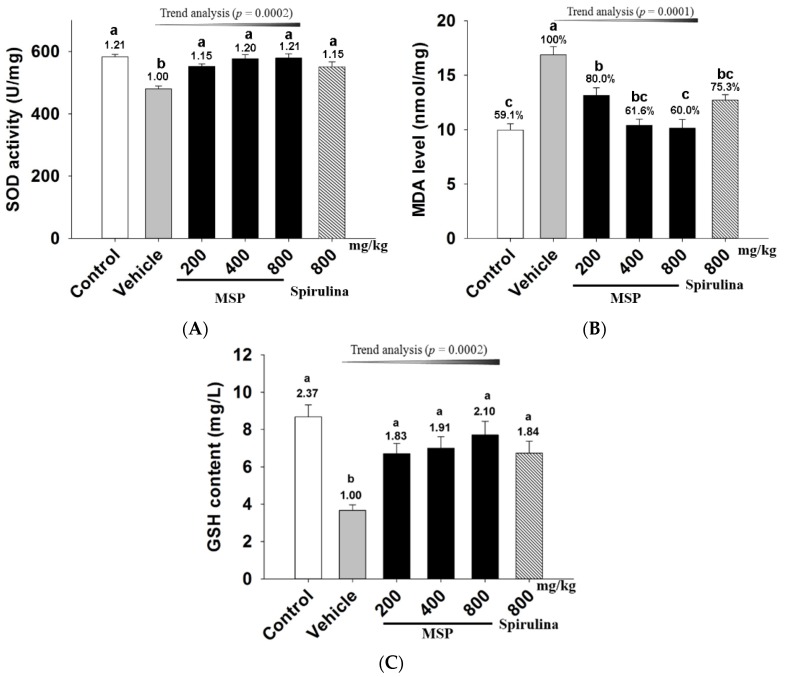
Effects of *Millettiae speciosae* Champ. total polysaccharides (MSP) on the plasma superoxide dismutase (SOD) activity (**A**); malondialdehyde (MDA) level (**B**) and glutathione (GSH) level (**C**) in mice after swimming for 30 min. Control group (open column), vehicle group (grey column), MSP-treated group (200, 400, 800 mg/kg/day, black column), spirulina group (800 mg/kg/day, twill column). Data are mean ±standard error of the mean (SEM) with *n* = 8 mice per group. Bars with different letters (a,b) are significant difference at *p* < 0.05. Numbers above the bars are the fold increase compared to the vehicle treatment.

To evaluate the antioxidant potential of MSP in mice, the blood levels of MDA, SOD, and GSH were measured after exhaustive exercise. As shown in Figure 5, forced swim (vehicle *vs.* control group, *p* < 0.05) significantly increased MDA (Figure 5-A), but decreased SOD (Figure 5-B) and GSH (Figure 5-C). In contrast, spirulina significantly increased SOD activity when compared to the vehicle treatment. Similarly, the three doses of MSP significantly up-regulated the SOD activity by 14.85%, 20.17%, and 20.67% respectively (Figure 5-A). Furthermore, MSP also dose-dependently decreased the MDA levels by 21.67%, 38.13%, and 39.69% respectively, (*p* < 0.01 *vs.* vehicle) (Figure 5-B). In addition, the GSH levels in the three doses of MSP groups were 6.70 ± 1.45, 7.00 ± 1.62, and 7.71 ± 1.92 mg/L, respectively, which were significantly up-regulated compared with the vehicle group.

### 3.5. Free Radical Scavenging Activity of MSP

The above results demonstrated that MSP have anti-oxidative actions *in vivo*. To further verify its effect, the free-radical scavenging activity of MSP was assessed by evaluating DPPH and ·OH free-radical scavenging activity *in vitro*. In Table 2, MSP showed a concentration-dependent ·OH radical scavenging activities. In addition, the activity of DPPH inhibition was dose-dependent. Vc, the positive control at the concentration of 200 μg/mL, had a radical scavenging effect similar to that of MSP. In contrast, the three concentrations of MSS showed no significant ·OH and DPPH radical scavenging activities.

### 3.6. Recommended Use of MSP for Human

The mouse MSP dose (800 mg/kg body weight) in this study can be converted to a human equivalent dose (HED) based on body surface area using the following formula from the US Food and Drug Administration: the conversion coefficient 12.3 was used to account for differences in body surface area between mice and humans as described previously [18]. In our study, a dose of 800 mg/kg in a mouse can be converted to 65 mg/kg in a 60 kg human.

**Table 2:** Effects of MSP and MSS on scavenging ·OH and DPPH radicals.

Group	Concentration (μg/mL)	HSR (%)	DSR (%)
Vc	200	93.57 ± 1.42	96.71 ± 0.12
MSP	50	14.72 ± 0.31	15.64 ± 0.81
	100	90.16 ± 1.00	88.43 ± 1.70
	200	92.71 ± 2.63	93.14 ± 0.42
MSS	50	2.99 ± 0.57	2.29 ± 0.23
	100	11.68 ± 0.43	10.93 ± 0.78
	200	13.06 ± 0.58	13.00 ± 0.85

HSR: the ·OH scavenging rate; DSR: The DPPH scavenging rate; MSP: Total polysaccharides; MSS: Total supernatant.

## 4. Discussion

Our present study aimed to extend our previous work by identifying whether polysaccharides are the active anti-fatigue fraction of MSE. Firstly, we separated MSE into MSP and MSS, followed by examining their influence on the bodyweight of the mice. The result proved that both MSP and MSS had no effect on the bodyweight of the mice after treatment for 20 consecutive days.

Then we evaluated their anti-fatigue activities in mice by ESE. This test is considered to be the most effective method to assess endurance capacity for exercise performance and known to give a good reproducibility [19]. Our experimental results revealed that MSP dose-dependently postponed the time to exhaustion in mice, while all doses of MSS groups showed no significant differences compared with the control group. Thus, the results demonstrated for the first time that it was MSP rather than MSS that was responsible for the anti-fatigue effect of MSE.

Exercise-induced physical fatigue could also be evaluated by quantifying blood and muscular biochemicals that are related to energy metabolism, including blood Glu, urea nitrogen, and lactic acid, as well as muscular glycogen. It is well accepted that muscular exercise causes rapid ATP consumption, and energy deficiency is a critical precipitating factor to physical fatigue [20]. During moderate to high intensity exercise, Glu is primarily utilized to support energy requirements and soon exhausted, because it is expeditiously available and can be directly oxidized to supply ATP in the blood [21]. Moreover, continuous exercises often lead to hypoglycemia, and suppress the functioning of the central nervous system, and in turn, decrease continual exercising ability. Therefore, the amount of Glu in blood can be used to indicate the speed and degree of fatigue development [22]. Besides, during physical exercise, energy can also be derived from the breakdown of glycogen. As such, restoring muscle glycogen is beneficial to prolonged exercise [8,9,23]. On the other hand, the decreased levels of muscle glycogen are a sensitive indicator for fatigue.

When the body fails to derive energy from carbohydrates and fat, protein and amino acids become alternative sources for catabolic metabolism to meet the energy requirement. The established marker of protein and amino acids catabolism is BUN. Consequently, increased BUN commonly reflects the decomposition of protein, which will jeopardize the contractive strength of muscle and finally lead to fatigue. The less the body is capable of adapting for exercise tolerance, the more significant increase in the urea nitrogen level can be seen [24]. Our result indicated that MSP had a positive effect to maintain muscular glycogen, increase blood Glu, and reduce the accumulation of BUN. Such a regulation on energy metabolism is advantageous during prolonged exercise.

The level of Lac, the product of glycolysis, is one of the important indicators of exercise fatigue. During strenuous exercise, muscle produces a considerable amount of Lac through anaerobic glycolysis [25]. The accumulation of Lac leads to a reduction of pH in muscle tissue, induces fatigue, and hampers exercise performance [26]. As a result, a rapid removal of Lac has been found to ameliorate fatigue [27]. In this study, MSP treatment significantly decreased plasma Lac, indicating that MSP possibly inhibited the accumulation of Lac, thereby postponing the occurrence of fatigue.

To evaluate whether MSP protected cells from damage after forced swimming, plasma LDH and CK were measured. As cytosolic enzymes, both LDH and CK are physiologically abundant in the cells of organs, including heart, kidney, and skeletal muscles. However, during the process of intense exercise, excessive free radicals are generated, which lead to damage of myocyte and leakage of LDH and CK into plasma [28]. LDH is an important enzyme involved in anaerobic glycolysis and gluconeogenesis, which boosts the oxidation-reduction reaction between pyruvate and L-lactate [29]. One of the functions of CK is to synthesize phosphocreatine, a rapid source of energy in cells [30]. Therefore, plasma LDH and CK are typical biomarkers for evaluating pathology of the myocardial infarcts and acute renal failure [31]. In the present study, the levels of LDH and CK in MSP groups were significantly decreased as compared to the vehicle group. The results suggested that the anti-fatigue activity of MSP may possibly be due to its ability to protect cells from damage during intense exercise.

The above mentioned blood LDH and CK are indirect indicators of cell damage [32]. In contrast, lipid peroxidation directly reflects the oxidative damage of cell membrane, which also contributes to the pathophysiology of fatigue [33]. Increased blood MDA, decreased GSH and SOD activity are known to be positively correlated with fatigue [9]. MDA is the main product of membrane lipid peroxidation by free radicals [34]. On the other hand, SOD is an important antioxidant enzyme against reactive oxygen species [35]. Thus, the increased SOD activity along with declined MDA in MSP groups can mitigate oxidative stress and ameliorate exercise-induced fatigue [17]. Furthermore, GSH plays a crucial role against oxidative stress, which can turn toxic substances into innocuous products by scavenging the free radicals [36]. Hence, adequate GSH is essential for preventing oxidative damage. In this study MSP treatment significantly improved SOD activity, elevating GSH while decreasing MDA, these findings strongly indicate MSP was able to protect the cells from lipid oxidation.

To test whether MSP has a direct anti-oxidative effect, we also performed the *in vitro* ·OH or DPPH scavenging test. These methods are commonly utilized to evaluate the radical scavenging activity of plant extracts. MSP was shown to possess a radical scavenging effect. In contrast, the three concentrations of MSS showed no significant ·OH or DPPH scavenging activities. The results suggested that MSP possessed pronounced scavenging activity against ·OH and DPPH radicals. These results are consistent with the *in vivo* antioxidant activity of MSP.

## 5. Conclusions

Our present experimental results for the first time showed that total polysaccharides isolated from *Millettiae speciosae* Champ. possessed an anti-fatigue activity in mice. This activity might be mediated through increasing energy sources, reducing the accumulation of BUN and Lac, and protecting muscle tissue from the damage of free radicals. The antioxidant properties of MSP have been established as the underlying mechanism of action for its anti-fatigue activity. To sum, the current results have amply demonstrated the great potential of MSP as a functional food to combat fatigue and oxidative stress.
